# A global item-level normalization approach to compare profiles of motivation in medical students

**DOI:** 10.3389/fmed.2026.1815385

**Published:** 2026-06-24

**Authors:** Rita Matos Sousa, Isabel Maria Matos, Vitor Hugo Pereira

**Affiliations:** 1 2CA-Braga, Braga, Portugal; 2 Escola de Medicina da Universidade do Minho, Braga, Portugal; 3 Life and Health Sciences Research Institute (ICVS), School of Medicine, University of Minho, Braga, Portugal; 4 ICVS/3B's-PT Government Associate Laboratory, Guimarães, Portugal; 5 Unidade Local de Saúde de Braga, Braga, Portugal; 6 Centro Universitário Max-Planck (UniMAX), São Paulo, Brazil; 7 Centro Universitário de Jaguariúna (UniFAJ), São Paulo, Brazil

**Keywords:** medical students, motivation, profile, psychometry, regulation

## Abstract

**Background:**

Academic success and students’ well-being are influenced by several factors, with motivation being a critical element. Previous studies in the literature have typically focused on comparing variations in motivation levels across distinct demographic or curricular subgroups. However, the measurement of the relative magnitude of different motivational levels within the same cohort has been overlooked. The main reason for this apparent lack of interest in defining a motivational profile through comparisons of various motivation types (such as intrinsic motivation, identified regulation, introjected regulation, external regulation, and amotivation) lies mainly in the psychometric complexity, particularly when subscales differ in item count and variance.

**Objective:**

In this study, we aimed to compare the relative levels of different motivation types within a single cohort of medical students, using a global item-level normalization procedure that enables valid cross-subscale comparisons.

**Methods:**

We conducted a cross-sectional study involving 266 medical students. The scores of a validated motivation scale, consisting of 18 items and based on self-determination theory, were globally standardized to a common metric (z-scores). Then, subscales were reconstructed from the normalized items, and a repeated-measures ANOVA, followed by Bonferroni-corrected pairwise comparisons, was performed to measure differences across motivation types.

**Results:**

Significant differences were observed among the various types of motivation. Identified regulation was significantly higher than all other forms of motivation. Intrinsic motivation exceeded both controlled forms (introjected and external regulation) and amotivation. Amotivation was significantly lower than all other motivation types, while introjected and external regulation did not differ significantly, which is consistent with their adjacent positions on the self-determination theory continuum. This motivational profile appears to be consistent across the academic stages evaluated at the time of assessment, as no significant interaction was observed between motivation types and stages of training; further longitudinal studies are required to confirm its temporal stability.

**Conclusion:**

This global item-level normalization approach offers a psychometrically valid method for comparing motivation types. The results reveal that medical students show a profile dominated by autonomous regulation, particularly identified regulation, followed by intrinsic motivation. Controlled regulation is found in the mid-range, while amotivation remains low. These findings are relevant to designing and tailoring interventions that support autonomous motivation in medical curricula.

## Introduction

Motivation is a central determinant of medical students’ development, with well-established effects on academic achievement, professional identity formation, and psychological well-being. These outcomes are directly relevant to clinicians and medical educators, as they influence not only students’ performance but also their long-term engagement, resilience, and the quality of patient care. Therefore, understanding how motivation operates within medical training is essential for designing educational environments that support both learning and well-being (1–4).

Within the framework of self-determination theory (SDT), motivation is conceptualized not as a simple high-low construct but as a continuum reflecting differences in the quality of motivation (1, 5, 6). At one end of this spectrum lies autonomous motivation, which includes intrinsic motivation (engaging in an activity for inherent enjoyment) and identified regulation, where individuals act because they personally value the activity. In contrast, controlled motivation refers to behaviors driven by internal pressures, such as guilt (introjected regulation), or by external contingencies, such as grades or approval (external regulation). At the opposite end of the continuum lies amotivation, representing a lack of intention or perceived value in the activity (1, 7, 8).

SDT further posits that the quality of motivation is shaped by the satisfaction of three basic psychological needs: autonomy, competence, and relatedness. When these needs are supported in a learning environment, individuals are more likely to develop autonomous motivation, resulting in greater engagement, persistence, and well-being. Conversely, when these needs are not met, motivation may shift toward more controlled forms or even amotivation, with potential negative effects on both performance and psychological health (1).

Importantly, SDT emphasizes that the quality of motivation plays a more significant role in influencing behavior than the sheer quantity of motivation. Autonomous forms of motivation have been consistently associated with deeper learning, persistence, and well-being, whereas controlled forms and amotivation have been linked to less adaptive outcomes (9). However, the majority of studies in medical education focus on between-group comparisons, such as differences based on gender or academic year (10, 11, 12). While these approaches can be informative, they provide limited insight into the internal motivational structure of a given cohort, which would be critical to understand and ultimately intervene in motivational regulation within a particular group and/or institution.

Understanding the motivational profile within a single cohort is particularly relevant for medical schools, as it allows institutions to tailor educational strategies to meet the specific needs of their students rather than relying on generalized assumptions. However, this type of analysis has been hindered by important methodological challenges. Standard instruments used to measure motivation differ in the number of items, variance, and distribution across subscales, making direct comparisons of raw scores among motivational types problematic. As a result, assessing the relative magnitude of different forms of motivation within individuals requires appropriate standardization procedures to ensure valid comparisons (13, 14).

Given that motivation subscales derived from SDT vary in item count and distributional properties, a direct comparison of raw subscale means risks producing deceptive conclusions. To address this limitation, the present study applies a global, item-level normalization approach, in which all items from a validated motivation scale are standardized to a common metric before reconstructing the motivation subscales. This procedure enables direct, psychometrically valid comparisons across motivation types, facilitating an accurate assessment of students’ internal motivational balance. The authors hypothesized that, within a single cohort of medical students, autonomous forms of motivation would be more prevalent than controlled forms and amotivation, and that this motivational profile would remain relatively stable across different stages of training. Accordingly, the primary objective of this study was to compare the relative levels of different motivation types within this single cohort using the normalization approach, with a secondary objective of examining whether the motivational profile differs between students at the beginning of their training and those in later stages of medical education.

## Methods

### Study design

A cross-sectional study was conducted to assess the motivational profiles of medical students. Data were collected using a structured questionnaire based on a validated motivation scale grounded in SDT (14). The questionnaire was administered through an online survey platform, and participation was voluntary and anonymous. Students were invited to participate through institutional email and during in-person academic sessions, where access to the questionnaire was provided through direct links and QR codes.

### Setting and participants

The study was conducted at the School of Medicine of the University of Minho in Portugal, which offers a 6-year medical program. Approximately 900 students are enrolled across all academic years, with an annual intake of approximately 120 students. All enrolled medical students were eligible to participate in the study. A total of 266 students completed the questionnaire.

### Instrument

Motivation was assessed using a previously validated scale specifically designed for medical students based on self-determination theory (14). The instrument consists of 18 items divided into 5 subscales representing different types of motivation: intrinsic motivation (IM), identified regulation (EMID), introjected regulation (EMIR), external regulation (EMER), and amotivation (AMOT). Responses are recorded on a scale ranging from 0 to 10, with higher scores indicating stronger endorsement of each item. Each subscale is composed of a different number of items: IM comprises six items (items 1, 4, 7, 8, 12, and 15), EMID comprises three items (items 2, 13, and 16), EMIR comprises three items (items 5, 14, and 18), EMER comprises three items (items 6, 10, and 11), and AMOT comprises three items (items 3, 9, and 17), as described in the original validation study (15). This unequal item distribution across the subscales is a key reason for applying the global item-level normalization procedure described below.

### Global item-level normalization

To enable direct, psychometrically valid comparisons across motivation types, all subscales were placed on a common metric using a global item-level normalization procedure. This approach was adopted to address the methodological limitation inherent in comparing subscales with unequal numbers of items and potentially different variances.

First, responses to all 18 items were pooled into a single distribution. A global z-score transformation was then applied using the overall mean and standard deviation computed across all item responses. Each item was subsequently transformed into its globally standardized version (P1_gn, P2_gn, …, P18_gn).

Motivation subscales were reconstructed by computing the mean of their respective globally normalized items, resulting in standardized subscale scores: IM, EMID, EMIR, EMER, and AMOT. This procedure ensured that the observed differences between motivation types reflected substantive variation rather than being influenced by item count or scaling. This item-level approach was deliberately chosen over subscale-level standardization for a key psychometric reason: to avoid potential differences in between-subscale variance that may carry substantive meaning and could be lost in the analysis. By pooling all items before standardization, the global mean and standard deviation reflect the full distribution of individual item responses across all motivation types, thereby preserving the relative magnitude of differences between subscales. Alternative approaches, such as comparing raw subscale means or dividing each subscale by its own standard deviation, are problematic when subscales differ in item counts and distributional properties because they yield scores that are not on a common metric and may lead to spurious or misleading comparisons.

### Statistical analysis

Descriptive statistics were computed for all globally normalized motivation subscales. Differences between motivation types were examined using a repeated-measures analysis of variance (ANOVA), with motivation type specified as a within-subject factor. When the assumption of sphericity was violated, Greenhouse–Geisser corrections were applied. Bonferroni-adjusted pairwise comparisons were performed to examine differences between individual motivation types.

To examine whether motivational profiles differed by stage of training, a mixed (split-plot) repeated-measures ANOVA was performed, with motivation type as the within-subject factor and stage of training (first-year vs. later-year students) as the between-subject factor. The interaction between motivation types and stages of training was used to assess whether the relative differences between motivation types varied across groups.

All analyses were conducted using IBM SPSS Statistics, version 30.0. A statistical significance level of 5% was established for the analyses (*p* < 0.05).

## Results

### Participant demographic profile

A total of 266 medical students participated in the study. Of these, 95 were first-year students, and 171 were enrolled in later years of medical training.

The sample consisted predominantly of women, with 210 female students (78.9%) and 56 male students (21.1%). The mean age of the participants was 20.68 years (SD = 3.52), reflecting a relatively homogeneous cohort typical of undergraduate medical education (see Table 1).

**Table 1:** Demographic characteristics of the study participants.

Variable	Value
Total participants (*n*)	266
First-year students (*n*/%)	95/35.7%
Later-year students (*n*/%)	171/64.3%
Female participants (*n*/%)	210/78.9%
Male participants (*n*/%)	56/21.1%

### Analysis of motivation instrument

A repeated-measures ANOVA was conducted to compare the five types of motivation: IM, EMID, EMIR, EMER, and AMOT, using subscales reconstructed from globally normalized item scores. Internal consistency of each subscale was assessed before normalization using Cronbach’s alpha: IM (*α* = 0.889), EMID (*α* = 0.79), EMIR (*α* = 0.754), EMER (*α* = 0.709), and AMOT (*α* = 0.810), indicating acceptable to good reliability across all subscales. To illustrate the added value of the normalization procedure, the ordinal ranking of the motivation types was also verified using the raw (unstandardized) subscale means. The ranking was preserved (EMID > IM > EMIR > EMER > AMOT); however, the raw means can obscure meaningful differences in relative magnitude due to the varying number of items in each subscale, underscoring the importance of the global normalization approach for valid cross-subscale comparisons.

Mauchly’s test indicated that the assumption of sphericity was violated, W = 0.488, *χ*^2^(9) = 188.77, *p* < 0.001. Accordingly, Greenhouse–Geisser corrections were applied (*ε* = 0.764). The analysis revealed a highly significant effect of motivation type, *F*(3.06, 810.33) = 465.65, *p* < 0.001, with a large effect size (partial *η*^2^ = 0.637).

**Figure 1:**
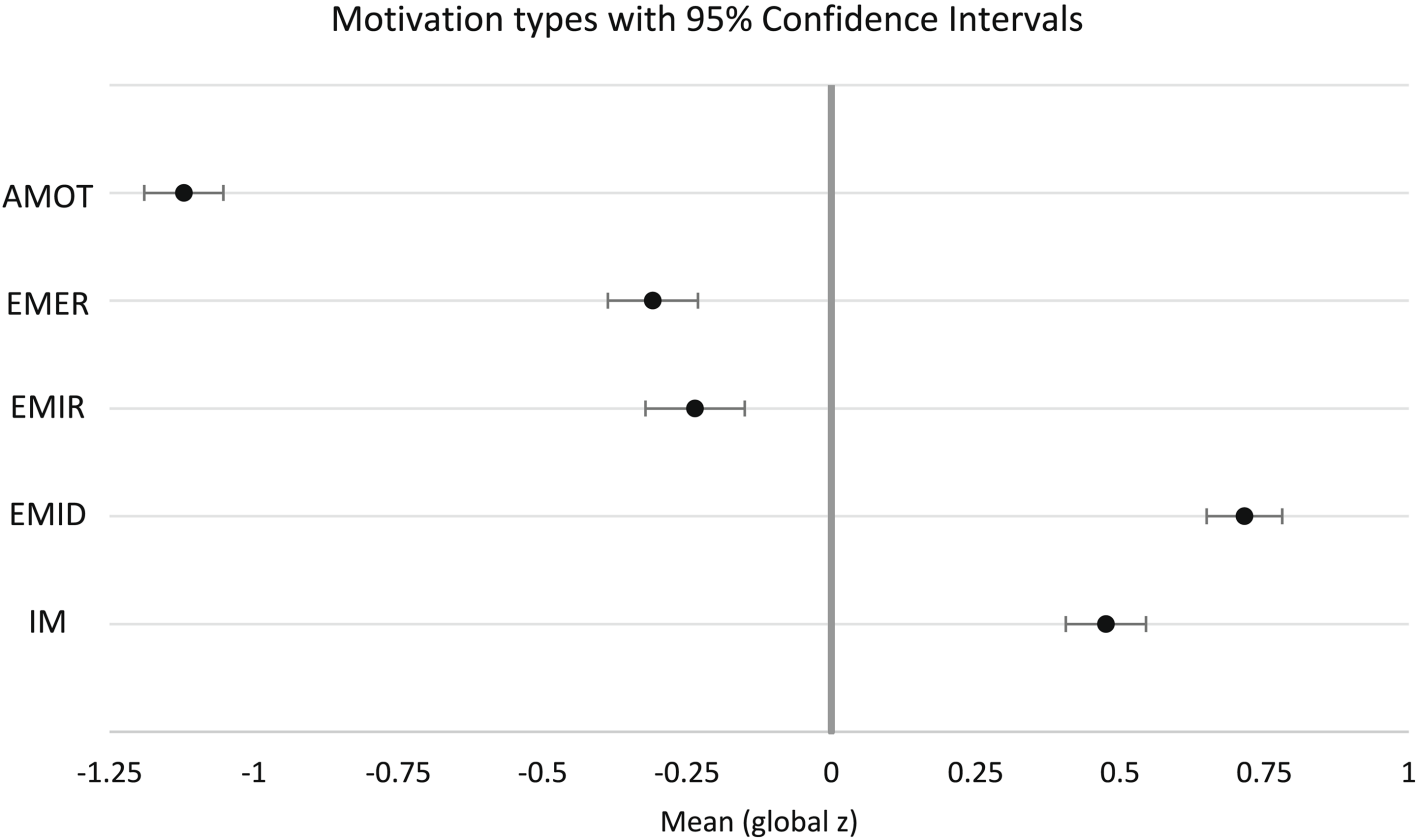
Estimated marginal means and 95% confidence intervals of globally normalized motivation scores for intrinsic motivation, identified regulation, introjected regulation, external regulation, and amotivation.

Estimated marginal means demonstrated a clear and theoretically coherent pattern across motivation types. EMID showed the highest mean level (M = 0.716), followed by IM (M = 0.476). EMIR (M = −0.236) and EMER (M = −0.309) occupied an intermediate position on the motivational continuum, while AMOT was markedly lower than all other types (M = −1.121) (Table 2; Figure 1).

**Table 2:** Descriptive statistics of the globally normalized motivation subscales.

Motivation type	Mean (global *z*)	SD	95% CI
IM	0.476	0.575	[0.406, 0.545]
EMID	0.716	0.539	[0.650, 0.781]
EMIR	−0.236	0.712	[−0.322, −0.150]
EMER	−0.309	0.647	[−0.387, −0.231]
AMOT	−1.121	0.568	[−1.190, −1.053]

Bonferroni-adjusted pairwise comparisons indicated that all motivation types differed significantly from one another (*p* < 0.001), except for EMIR and EMER, which did not differ significantly (*p* = 0.782). Specifically, EMID was significantly higher than IM, the two controlled forms of regulation, and AMOT. IM was significantly higher than EMIR, EMER, and AMOT. Both controlled forms of regulation were significantly higher than AMOT (Table 3).

**Table 3:** Bonferroni-adjusted pairwise comparisons between the globally normalized motivation types.

Comparison	Mean difference	*p* (Bonferroni)
IM vs. EMID	−0.240	<0.001
IM vs. EMIR	0.712	<0.001
IM vs. EMER	0.785	<0.001
IM vs. AMOT	1.597	<0.001
EMID vs. EMIR	0.952	<0.001
EMID vs. EMER	1.025	<0.001
EMID vs. AMOT	1.837	<0.001
EMIR vs. EMER	0.073	0.782
EMIR vs. AMOT	0.885	<0.001
EMER vs. AMOT	0.812	<0.001

### Motivational profiles by stages of training

A mixed repeated-measures ANOVA was conducted to examine whether the relative differences in motivation types varied between first-year students and students in later years of medical training. Mauchly’s test indicated that the assumption of sphericity was violated; therefore, Greenhouse–Geisser corrections were applied (see Table 4).

**Table 4:** Descriptive statistics of globally normalized motivation types by stage of training.

Motivation type	First year (*n* = 95) Mean (SD)	Later years (*n* = 162) Mean (SD)
IM	0.337 (0.65)	0.540 (0.52)
EMID	0.598 (0.62)	0.764 (0.48)
EMIR	−0.259 (0.77)	−0.255 (0.67)
EMER	−0.330 (0.68)	−0.298 (0.64)
AMOT	−1.136 (0.60)	−1.106 (0.56)

**Figure 2:**
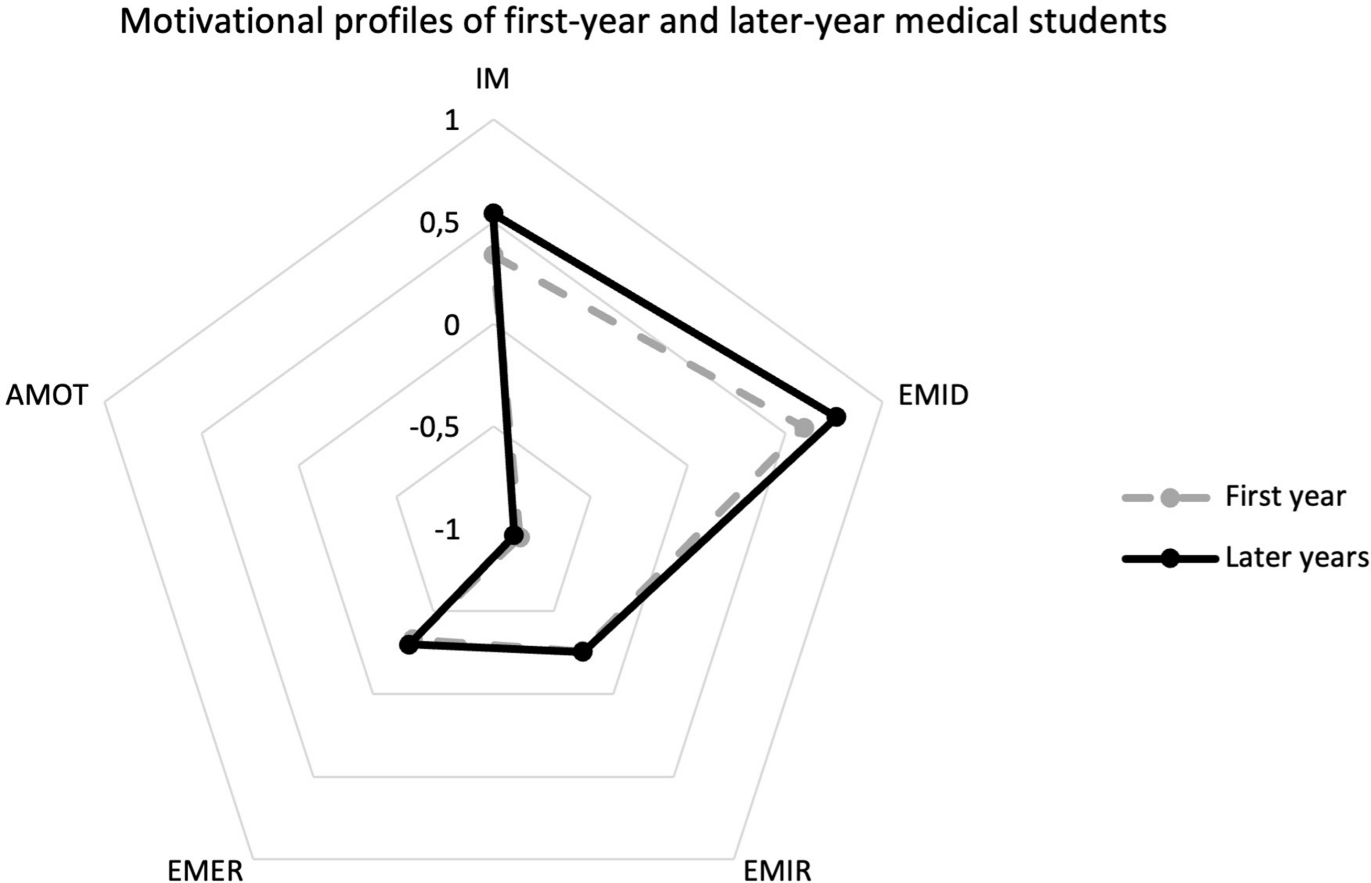
Motivational profiles of first-year students (gray line) and later-year students (black line). No significant differences were found between these profiles, indicating cross-sectional consistency of the motivational profile across academic stages.

A significant main effect of motivation type was observed, *F*(3.05, 776.38) = 396.41, *p* < 0.001, partial *η*^2^ = 0.609, indicating robust differences between motivation types across the sample. The interaction between motivation types and stages of training was not significant, *F*(3.05, 776.38) = 1.62, *p* = 0.183, partial *η*^2^ = 0.006, indicating that the relative differences between motivation types were comparable in first-year students and students enrolled in later years (Figure 2). Descriptively, later-year students showed slightly higher levels of autonomous motivation (IM and EMID) than first-year students; however, the overall motivational pattern remained stable across training stages.

## Discussion

The results presented herein provide a comparison of the distinct motivation types among medical students, which is relevant to mitigating the existing gap in the literature. This comparison is possible as this study has used an appropriate methodology based on global item-level normalization. This approach places all motivation subscales on a common metric dimension, enabling correct comparisons between the scores of various subscales. It also circumvents the limitations triggered by the assumption that such comparisons are legitimate when using only raw data or unstandardized averages. By doing so, we offer a clearer perspective on the internal motivational structure of medical students.

The present findings reveal that identified regulation is the most prominent form of motivation in this cohort of medical students. Within the framework of SDT, this form of motivation reflects a high degree of internalization among students and is consistently associated with adaptive learning behaviors and sustained engagement (1, 16–19). This finding suggests that training in medicine is perceived and highly valued by students, as it aligns with their future professional careers. Consistently, with a high degree of internalization, the present study also reveals that intrinsic motivation was relatively high. This profile is likely a result of the strong sense of purpose and commitment of medical students, which coexists with enjoyment and interest within the highly demanding context of medical education. In fact, the balanced scores of identified regulation and intrinsic motivation reflect the mental compromise between the commitment to becoming a clinician and the perceived relevance of their professional identity (4, 20, 21).

On the other hand, controlled forms of motivation, such as introjected regulation and external regulation, displayed a less prominent position in the motivational profile of the medical students in this cohort. Additionally, there were no significant differences between these two forms of motivation, which is theoretically coherent, as these two forms of regulation are adjacent on the SDT continuum and represent partially internalized but still controlled motivational processes (1). This observation suggests that internal pressures (e.g., exigencies of self-confidence or feelings of guilt) and external demands (e.g., assessments or expectations) may operate in tandem in terms of motivation for medical students. Finally, the component of amotivation was remarkably lower than all other motivation types, showing that intentional engagement is present in the majority of medical students in this cohort, which is a positive sign, as amotivation has been consistently associated with disengagement, poorer academic outcomes, and increased psychological distress (22).

An important extension of these findings concerns the comparison between first-year students and those in later years of training. When analyzed separately, both groups exhibited the same ordering of motivational types, and no significant interaction was observed between motivational type and stage of training. This suggests that the relative differences among intrinsic, identified, controlled, and amotivated forms of regulation are already established at the beginning of medical school and remain largely consistent across academic stages in cross-sectional comparisons; longitudinal research is needed to determine whether this pattern persists within the same individuals over time. At the same time, descriptive differences suggested that later-year students displayed slightly higher levels of autonomous motivation, particularly intrinsic motivation and identified regulation. This pattern aligns with SDT, which conceptualizes motivation as a dynamic process of internalization occurring within a relatively stable motivational framework (1, 23). As students progress through training, increased clinical exposure and professional identity consolidation may strengthen autonomous motivation without fundamentally altering the overall motivational profile.

This study contributes comparable data on motivation types to understand their relative prominence and inform curriculum design and educational practice. The predominance of autonomous forms of motivation herein observed is positive, as this type of motivation has been associated with positive outcomes, such as deeper learning strategies, greater persistence, reduced burnout, and improved psychological well-being (24–26). However, this should be strengthened by educational approaches to prevent the decline of such motivation among medical students. This educational support may assume distinct forms, including autonomy-supportive teaching practices by offering meaningful choices and acknowledging students’ perspectives or promoting relevance-based learning by explicitly linking curricular content to clinical practice. Moreover, these efforts should be accompanied by interventions that reduce excessive external pressures, particularly those that may undermine autonomy without contributing educational value. Strategies should be implemented to identify and support amotivated students, who may be at an increased risk for disengagement or distress. In fact, to maintain sustained engagement in the challenging context of medical training, medical educators should align instructional strategies with the motivational profiles of their students. Unlike much of the existing literature, including previous studies by the authors, the present study does not focus on between-group comparisons based on demographic or curricular variables but rather on determining the motivational profile of students by providing a psychometrically robust analysis of motivational types.

### Limitations

Several limitations should be acknowledged. First, the study was conducted within a single medical school, which may limit the generalizability of the findings to other educational contexts. Second, the cross-sectional design precludes conclusions regarding causal relationships or changes in motivation over time. Third, the use of self-reported measures introduces the possibility of response bias. Finally, participation was voluntary, which may have resulted in selection bias. In particular, the cross-sectional design does not allow for inferences about motivational stability over time within the same individuals; the observed consistency between first-year and later-year students reflects a between-group comparison at a single time point and should not be interpreted as evidence of longitudinal stability. Future longitudinal studies tracking the same cohort across academic years would be necessary to confirm whether the motivational profile remains stable within individuals throughout medical training. Additionally, the Cronbach’s alpha values and raw score comparisons reported herein are based on the observed data of this specific sample and should be interpreted in the context of this study’s setting and sample size.

## Conclusion

In conclusion, the application of global item-level normalization to this dataset provides a powerful method for comparing motivational types within individuals and for determining a motivational profile of each student. In this cohort, medical students demonstrate a motivational profile dominated by identified regulation and intrinsic motivation, with controlled forms in the mid-range and amotivation at the lowest end. Such a result should inform medical schools to design precise and customized strategies that sustain and, if possible, enhance autonomous motivation.

## Data Availability

The raw data supporting the conclusions of this article will be made available by the authors, without undue reservation.
